# Diagnosing abdominal neoplasms using a T2 mapping radial turbo spin-echo technique with partial volume correction

**DOI:** 10.1007/s00330-025-11931-4

**Published:** 2025-08-30

**Authors:** Mahesh B. Keerthivasan, Brian Toner, Jean-Philippe Galons, Kevin Johnson, Ali Bilgin, Diego R. Martin, Maria I. Altbach

**Affiliations:** 1 Department of Radiology and Imaging Sciences, University of Arizona, Tucson, Arizona USA; 2 MR R&D Collaborations, Siemens Medical Solutions USA, New York New York, USA; 3 Applied Mathematics Program, University of Arizona, Tucson, Arizona USA; 4 Department of Biomedical Engineering, University of Arizona, Tucson, Arizona USA; 5 Department of Electrical and Computer Engineering, University of Arizona, Tucson, Arizona USA

**Keywords:** Abdominal imaging, T2 mapping, Partial volume effects, Radial turbo spin echo

## Abstract

**Objective:**

T2 mapping allows for the classification of focal liver lesions, differentiating malignancies from the most common benign liver lesions, hemangiomas, and bile duct hamartomas (BDH). Partial volume (PV) due to the presence of liver and lesion within the same voxel confounds the classification of small lesions. Our objective is to develop a robust two-component T2 estimation technique (SEPG2-SP) to enable accurate T2 estimation in the presence of PV.

**Materials and methods:**

T2 estimation accuracy was evaluated using computer simulations, physical phantom data, and in vivo in 27 subjects with focal liver lesions (16 males, 62.4 ± 14.3 years old; 11 females, 66.8 ± 5.8 years old) imaged at 1.5 T with a radial turbo spin-echo (RADTSE) technique. The SEPG2-SP model was compared to a single-component model, which does not account for PV. The area under the receiver operator characteristic curve (AUROC) was used to analyze lesion classification.

**Results:**

Phantom data showed that the SEPG2-SP model had a T2 estimation error of 2–9% while the single component model had a larger error of 9–23%. Analysis of in vivo data from 68 focal liver lesions (33 malignancies, 7 hemangiomas, and 28 BDH) showed that the SEPG2-SP model classified all lesions correctly (AUROC = 1), regardless of their size. On the other hand, with the single-component model, there was overlap between malignancies and benign lesions driven by misclassification of hemangiomas as malignancies (AUROC = 0.84).

**Conclusions:**

The two-component T2 model improved the characterization of focal liver lesions affected by PV, yielding complete separation of malignancies from the most common benign liver lesions.

**Key Points:**

***Question*** *Partial volume effects result in T2 estimation errors that confound the classification of small focal liver lesions.*

***Findings*** *The proposed two-component T2 estimation technique improves T2 estimation accuracy and allows accurate characterization of focal liver lesions in the presence of partial volume.*

***Clinical relevance*** *The T2 mapping technique described here offers a practical and reliable approach for quantitatively classifying focal liver lesions. It enables differentiation between the most common benign liver lesions and malignancies, even in small tumors impacted by partial volume effects.*

**Graphical Abstract:**

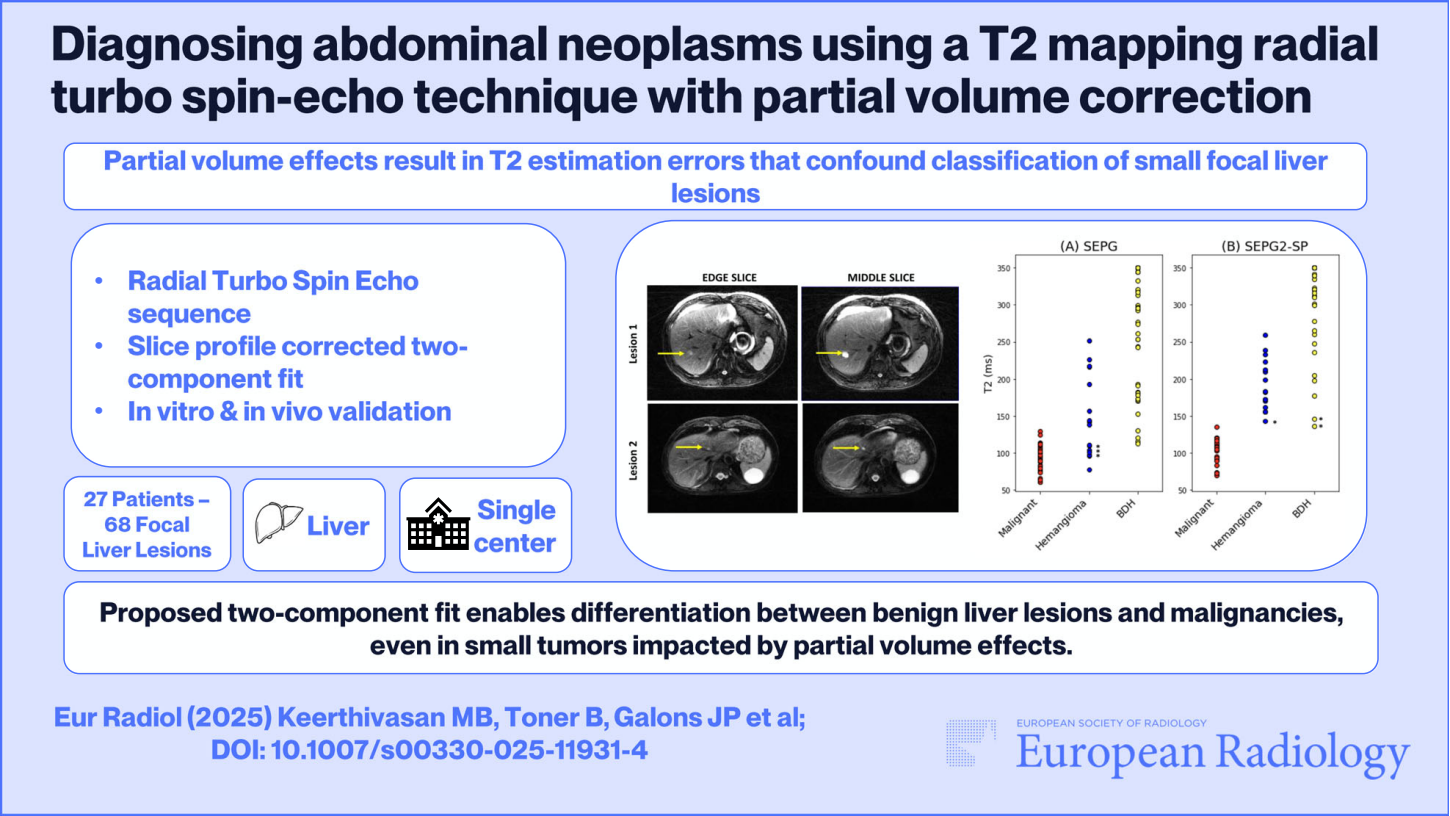

## Introduction

T2-weighted imaging is an essential part of a comprehensive abdominal MRI, frequently used to diagnose conditions such as focal liver lesions. These are typically diagnosed as benign or malignant based on the qualitative assessment of signal intensity relative to liver [1, 2]. T2 relaxation has also been proposed for the quantitative characterization of abdominal neoplasms. Previous studies have found significant differences in T2 relaxation times between different types of focal liver lesions [3–8], providing a range of classification for malignancies, hemangiomas and cysts, mostly representing bile duct hamartomas (BDH) [9].

To obtain accurate T2 estimates, images are acquired at different echo times (TE) to sample the T2 decay curve. T2 mapping techniques based on conventional (i.e., Cartesian) multi-echo spin-echo [10], turbo spin echo (TSE) [11], T2-prepared gradient echo [12], single-shot fast spin-echo [13], or echo planar imaging [7, 13] typically suffer from motion averaging, low spatial and/or temporal resolution, mis-registered TE images, or long acquisition times.

Radial turbo spin echo (RADTSE)-based methods have been proposed for quantitative T2 mapping [5, 6, 11, 14–16]. RADTSE yields high spatiotemporal resolution and allows for the reconstruction of images at multiple TEs (which are registered by the nature of the acquisition) from a short acquisition (i.e., a breath hold). The radial trajectory makes the technique robust to motion, making the technique better suited for abdominal imaging [6].

In breath-held abdomen TSE imaging, slice coverage is limited by the specific absorption rate (SAR) and requires the use of refocusing pulses with flip angles less than 180°. Imperfections in the refocusing slice profile lead to the generation of indirect echoes, which modulate the signal evolution and cause it to deviate from the ideal single exponential model. Techniques based on slice resolved extended phase graphs (SEPG) [17] and Bloch equations [18, 19] have been proposed to accurately estimate T2 in the presence of indirect echoes. When these are incorporated in model-based reconstructions of undersampled RADTSE data, accurate T2 estimation is achieved regardless of the flip angle used in the train of refocusing pulses [6, 15].

A critical issue for the characterization of small liver lesions based on qualitative evaluations or quantitative parameter estimation is the partial volume (PV) effect due to the presence of liver and lesion within the same voxel. When using T2 values to characterize focal liver lesions, PV may cause underestimation of the lesion T2 due to the lower T2 of liver, versus the higher T2 of a lesion [20]. This underestimation may result in false positive misclassification of a small hemangioma or BDH as a malignancy, which characteristically has lower T2 values compared to BDH or hemangiomas [3].

A multi-component T2 estimation technique [21] was previously proposed for the characterization of abdominal lesions in the presence of PV. This approach was based on a biexponential model for T2 decay, rather than the more accurate SEPG or Bloch models and thus, did not consider the effect of RF pulse imperfections and slice profile variations in T2 estimation. In particular, the method is limited to using 180° refocusing RF pulses, which limits echo train length and slice coverage due to SAR. Multi-component models have also been proposed for more accurate T2 estimation in applications where signal contribution from more than two components occurs within the voxel [22, 23]. While some of these models incorporate the effect of indirect echoes resulting from inhomogeneous B1+ in TSE acquisitions [23–26], they do not consider slice profile variations that affect the refocusing flip angles when using a selective 2D excitation (in particular when the refocusing flip angle is less than 180°). Instead, thicker refocusing slices [27] or non-selective refocusing have been employed to ensure uniform distribution of flip angles across the excited slice. However, with 2D multi-slice imaging protocols that employ slice gaps, partial volume effects and the lesion’s location with the excited slice profile affect the observed signal.

In this work, we present a two-component model for accurate T2 estimation to solve for PV and provide superior classification of liver tumors. The proposed technique models the effect of RF pulse slice profile imperfections and generalizes across both constant and variable refocusing pulse flip angles. The technique is evaluated in the RADTSE sequence, and estimation performance is validated using numerical simulations and model phantoms. Performance of the sequence is demonstrated in vivo for the characterization of abdominal neoplasms using PV-corrected T2 estimates.

## Materials and methods

Informed consent was obtained prior to imaging human subjects in compliance with the university’s Institutional Review Board requirements.

### Signal modeling

In the absence of PV, the observed signal decay from a turbo spin echo sequence can be accurately represented by the extended phase graph model (EPG) proposed by [28]. The EPG model considers T2 and T1 relaxation as well as the effect of B1+ inhomogeneity. To account for slice profile imperfections, we use the SEPG model, where the EPG signal is integrated over the slice profile [21] as explicitly described in Equation S1 in Supplementary Material. The SEPG model accounts for the coherent pathways the spins experience as they are subjected to the various RF pulses and accurately represents the signal evolution along the echo train even in the presence of B1+ inhomogeneities and slice profile imperfections.

**Fig. 1:**
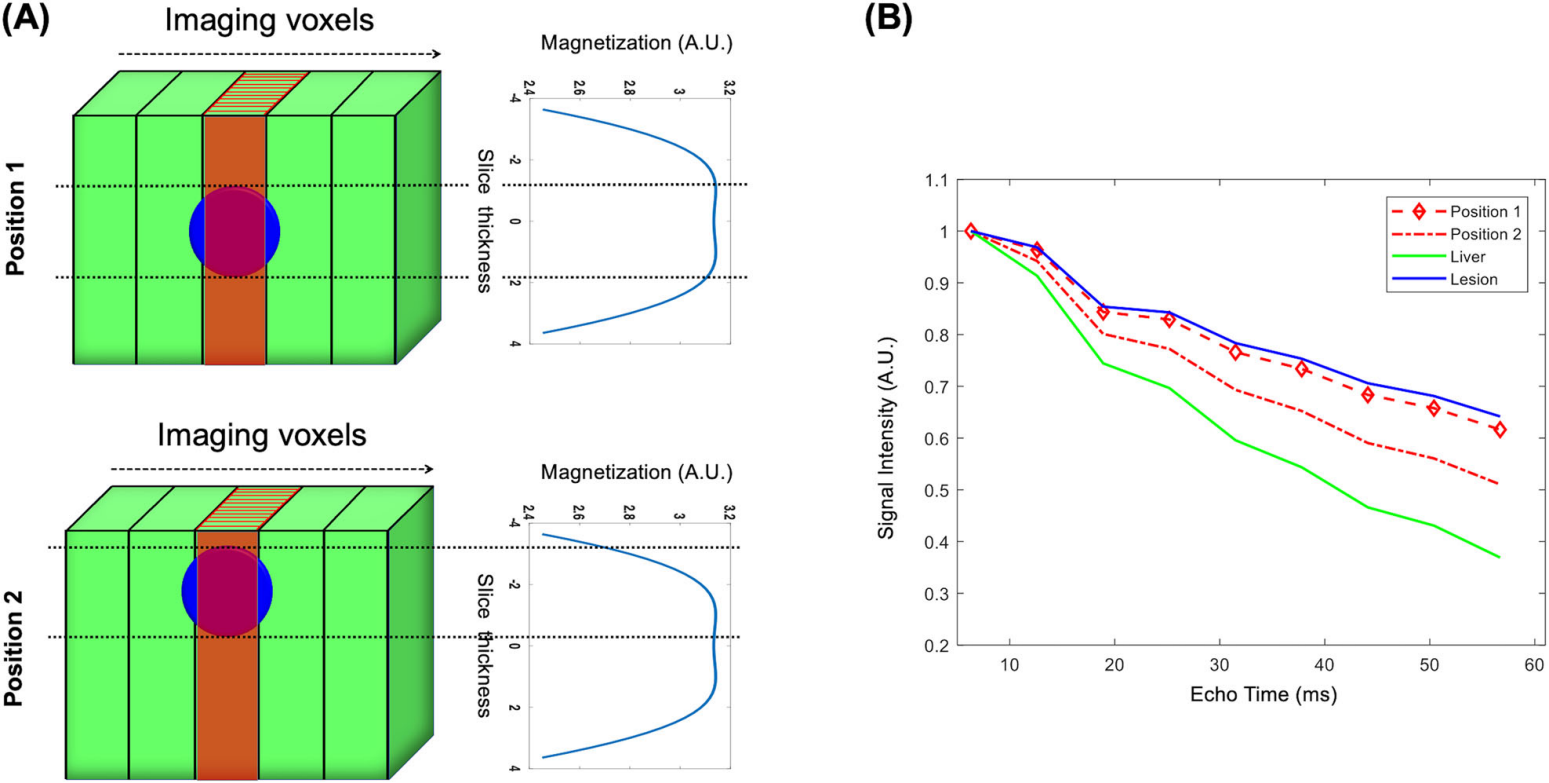
**A** Illustration of slice profile effects on two-component parameter estimation. Assuming a lesion (blue circle) embedded within background tissue (green), flip angle variations across the excited/refocused slice cause the two tissue types to experience different coherent pathways. **B** The observed signal from the slice is a cumulative sum of signal from these two components, which depends on the slice profile and the position of the lesion within the slice. When the lesion is at the center of the slice, the signal decay (red curve—position 1) is closer to the decay of the lesion without PV (blue curve). When the lesion is at the edge of the slice, the signal (red curve—position 2) has an intermediate decay between lesion (blue curve) and liver (green curve) due to partial volume

When there are two tissue types within a slice, such as it occurs when imaging a small tumor embedded in background liver tissue, the observed signal from the slice must include the contribution of the two tissue components. In the case of focal liver lesions, the RF slice profile affects signal from lesion and background differently (Fig. 1A) depending on the location of the lesion within the excited slice. This causes the two tissue components to experience different coherent pathways and thus different T2 decay curves (Fig. 1B). When the lesion is at the center of the slice, the signal decay (red curve—position 1) is closer to the decay of the lesion without PV (blue curve). When the lesion is at the edge of the slice, the signal (red curve—position 2) has an intermediate decay between lesion (blue curve) and liver (green curve) due to PV.

To account for the differences in slice profiles experienced by lesion and background, we can decompose the slice profile into two spatial sub-profiles and the observed signal is obtained by integrating the signal from each component over the sub-profiles. The proposed two-component SEPG model with slice profile variation (SEPG2-SP) is described in Equation S4 in Supplementary Material.

T2 estimation for the two-component model is performed using the joint fitting framework [21] described in Supplementary Material S2.

### Numerical simulations

Monte Carlo simulations were conducted to evaluate the performance of the SEPG2-SP model. The details of these simulations are described in Supplementary Material S3.

### Imaging experiments

Data were acquired on 1.5-T Aera and 3.0-T Skyra scanners (Siemens Healthineers). Data were acquired with two variants of the prototype RADTSE pulse sequence: RADTSE-CFA with constant refocusing flip angles (CFA) and RADTSE-VFA with variable refocusing flip angles (VFA) [6]. All image reconstructions and processing were performed offline using MATLAB (MathWorks).

#### Phantom imaging

A physical phantom was prepared to evaluate the effect of PV in T2 estimation. The phantom consisted of spherical bulbs with diameters of 8 mm, 9.5 mm and 12 mm filled with a 0.14 mM gadobenate dimeglumine (Multihance, Bracco Diagnostic Inc) solution to represent small hemangiomas (T2 = 160 ms, T1 = 1090 ms). The bulbs were immersed in a container filled with a 3.6 mM gadobenate dimeglumine solution (T2 = 44 ms, T1 = 60 ms) to represent spherical lesions embedded in background tissue. Details of the phantom experiments are given in Supplementary Material S4.

#### In vivo imaging

The RADTSE-CFA and/or RADTSE-VFA pulse sequences were added to the clinical abdomen-pelvis protocol at 1.5 T, and breath-held data were acquired prospectively on patients when the clinical schedule permitted it. The pulse sequence acquisition parameters are listed in Table 1.

**Table 1:** MRI acquisition parameters for the constant and variable flip angle radial turbo spin echo sequences used in this study

Parameters	RADTSE-CFA	RADTSE-VFA
Field of view (cm)	36–42	36–42
Base resolution	256	256
Radial views	192	192
Resolution (mm^2^)	1.4 × 1.4–1.64 × 1.64	1.4 × 1.4–1.64 × 1.64
Slice thickness (mm)	8	8
Readout bandwidth (Hz/Pixel)	501	501
Refocusing flip angle (deg)	150°	[70°, 100°, 40°, 130°]
Preparation pulses	Spectral Attenuated Inversion Recovery	Spectral Attenuated Inversion Recovery
Echo spacing (ms)	7.1	7.1
Repetition time (ms)	3000	3000
Number of slices	21	21
Scan time	3 breath-holds	3 breath-holds

Variable flip angles were generated based on the scheme described in [6]

*RADTSE-CFA* constant flip angle radial turbo spin echo, *RADTSE-VFA* variable flip angle radial turbo spin echo

From the 130 clinical patients scanned with RADTSE between 2014 and 2018, 27 (16 males 62.4 ± 14.3 years old, 11 females 66.8 ± 5.8 years old) had prescribed slices containing untreated focal liver lesions according to the clinical radiological report. 21 subjects were scanned with RADTSE-CFA and 6 with RADTSE-VFA. This dataset yielded 68 focal liver lesions to test the proposed two-component T2 mapping technique: 33 malignancies (4 hepatocellular carcinoma, 29 metastases) and 35 benign lesions (7 hemangiomas, 28 BDH). The diameter of the lesions ranged from 5.0 to 60 mm, with 17 lesions ranging from 5.0 to 10.0 mm, 14 lesions ranging from 10.2 to 15.0 mm, 14 lesions ranging from 15.1 to 20.0 mm, and 25 lesions > 20.0 mm.

The acquired radial data were reconstructed using an iterative reconstruction algorithm [6], and corresponding T2 maps were generated. The details of the image reconstruction are provided in Supplementary Material S2.

### Lesion analysis

Lesions were characterized as part of the clinical evaluation by trained radiologists using conventional T2-weighted, T1-weighted, and dynamic contrast-enhanced images and/or after follow-up. ROIs were manually placed around the focal liver lesions using the RADTSE T2-weighted images corresponding to TE ~ 90 ms. Since the RADTSE T2-weighted images and T2 maps are perfectly registered, T2 values were extracted by applying the ROIs to the corresponding T2 maps. A receiver operating characteristic (ROC) analysis was performed to analyze the ability of the SEPG and SEPG2-SP methods to classify lesions. The area under the ROC curve (AUROC) was used as the classification metric.

## Results

### Numerical simulations

Results of the Monte Carlo simulations evaluating the effect of PV on T2 estimation are shown in Table 2 for constant and variable flip angle refocusing schemes. Table 2A shows the effect on T2 estimation for the SEPG and SEPG2-SP models when (i) changing the position of an 8-mm diameter lesion within a 10-mm thick slice and (ii) changing the diameter of a lesion at the center of a 10-mm thick slice. The proposed SEPG2-SP model corrects for the combined effect of partial volume and imperfect slice profile. Note that the SEPG model has large estimation errors reaching ~50% for an 8-mm diameter lesion offset by 6 mm from the center of the slice. The error is reduced to ≤ 5.6% with the proposed two-component SEPG2-SP model, with T2 accuracy improving as the lesion moves toward the center of the slice, where the slice profile is more uniform. The estimation error with both the SEPG and SEPG2-SP models was reduced for lesions centered within thinner slices, as shown in Table 2B (simulations within an 8-mm thick slice) and Table 2C (simulations within a 6-mm thick slice). However, as the lesion is offset from the center, the T2 estimation error with SEPG can be large even for thinner slices (e.g., 56% for an 8-mm diameter lesion within an 8-mm thick slice and 27% for the same lesion within a 6-mm thick slice). With the SEPG2-SP model, the corresponding T2 errors are ≤ 9.6% and ≤ 2.1%, respectively.

**Table 2:** Results of Monte Carlo simulations comparing T2 estimation accuracy of the single-component SEPG model and the two-component model with slice profile variation, SEPG2-SP

Lesion offset (mm)	SEPG	SEPG2-SP	Lesion diameter (mm)	SEPG	SEPG2-SP
**A: Lesion within a 10-mm thick slice**					
**Relative error in T2—Constant flip angle refocusing scheme**					
6	43.7%	3.9%	6	19.2%	5.0%
4	27.1%	3.3%	8	7.9%	1.7%
2	8.9%	3.3%	10	2.4%	2.2%
0	1.1%	1.7%	12	0.9%	1.1%
**Relative error in T2—Variable flip angle refocusing scheme**					
6	42.3%	5.6%	6	20.1%	1.7%
4	26.5%	2.8%	8	8.3%	1.1%
2	8.6%	2.2%	10	2.2%	2.8%
0	1.2%	1.7%	12	0.9%	0.6%

**B: Lesion within an 8-mm thick slice**					
**Relative error in T2—Constant flip angle refocusing scheme**					
6	56.1%	5.1%	6	9.4%	2.1%
4	32.4%	4.2%	8	1.9%	1.6%
2	11.1%	3.1%	10	1.2%	1.3%
0	0.8%	1.0%	12	0.9%	0.9%
**Relative error in T2—Variable flip angle refocusing scheme**					
6	55.2%	4.9%	6	9.6%	9.4%
4	31.9%	4.4%	8	1.7%	1.5%
2	11.3%	3.1%	10	1.3%	1.1%
0	1.0%	0.9%	12	1.0%	1.1%

**C: Lesion within a 6-mm thick slice**					
**Relative error in T2—Constant flip angle refocusing scheme**					
6	26.8%	3.9%	6	2.1%	1.9%
4	14.3%	3.6%	8	1.2%	0.9%
2	2.8%	1.9%	10	0.9%	1.0%
0	0.8%	0.8%	12	0.8%	0.9%
**Relative error in T2—Variable flip angle refocusing scheme**					
6	27.1%	3.9%	6	1.9%	2.0%
4	14.7%	3.4%	8	1.1%	1.1%
2	2.5%	1.7%	10	1.0%	0.8%
0	0.8%	0.7%	12	0.9%	1.0%

The simulations show the effect of (i) changing the position of an 8-mm diameter spherical lesion within the slice and (ii) changing the diameter of a lesion centered within the slice. Simulations were performed for constant flip angle and variable flip angle refocusing RF schemes for (**A**) a 10-mm thick slice, (**B**) an 8-mm thick slice, and (**C**) a 6-mm thick slice. In the simulations, the true liver T2 was 40 ms, and the true lesion T2 ($T{2}_{Ref}$) was 180 ms representing a benign lesion. The relative error is defined as $Relative\,Error\,({{{\rm{ \% }}}})=\frac{|T{2}_{Ref}-T{2}_{Est}|}{T{2}_{Ref}}\ast 100$

*SEPG* slice resolved extended phase graph model, *SEPG2-SP* two-component slice resolved extended phase graph model with slice profile correction

### Phantom imaging

Table 3 shows the relative T2 error for the 8-mm, 9.5-mm, and 12-mm diameter spherical bulbs for three different relative slice positions for both RADTSE-CFA and RADTSE-VFA. With the SEPG2-SP model, the relative T2 error for all the spherical bulbs is below 6% when the bulb is at the center (0% offset) or at a 50% offset from the center of the acquired slice. When the bulb is at the edge of the slice (80% offset from the center of the acquired slice), the error is 8–9%. In contrast, the single-component SEPG model yields T2 errors ranging from 9 to 23%.

**Table 3:** Results of physical phantom experiments showing the effect of changing lesion position relative to the center of a 10 mm imaging slice and the lesion size on T2 estimation accuracy for (**A**) RADTSE with a constant flip angle (CFA) and (**B**) variable flip angle (VFA) refocusing RF schemes

Offset from center sphere (mm)	(A) Relative Error in T2—RADTSE-CFA					
	8 mm		9.5 mm		12 mm	
	SEPG	SEPG2-SP	SEPG	SEPG2-SP	SEPG	SEPG2-SP
6	23.1%	8.2%	19.6%	7.45%	12.7%	4.3%
4	8.9%	5.1%	6.7%	5.1%	3.9%	4.1%
0	1.8%	2.2%	1.8%	2.67%	2.1%	3.8%

Offset from center sphere	(B) Relative Error in T2—RADTSE-VFA					
	8 mm		9.5 mm		12 mm	
	SEPG	SEPG2-SP	SEPG	SEPG2-SP	SEPG	SEPG2-SP
6	22.4%	8.5%	20.1%	7.2%	12.1%	6.3%
4	9.3%	5.7%	6.9%	4.9%	4.2%	5.3%
0	1.8%	2.2%	1.8%	2.67%	2.1%	3.8%

Overall, T2 accuracy is higher for the SEPG2-SP compared to the SEPG model, which does not account for PV. T2 estimation accuracy is higher as the lesion moves toward the center of the slice (where the slice profile is more uniform) and as lesion size increases, since more of the lesion compartment experiences the true refocusing flip angle. The relative error is defined with respect to the single-echo spin echo reference ($T{2}_{SE}$) as: $Relative\,Error\,({{{\rm{ \% }}}})=\frac{|T{2}_{SE}-T{2}_{RADTSE}|}{T{2}_{SE}}\ast 100$

*SEPG* slice resolved extended phase graph model, *SEPG2-SP* two-component slice resolved extended phase graph model with slice profile correction, *RADTSE-CFA* constant flip angle radial turbo spin echo, *RADTSE-VFA* variable flip angle radial turbo spin echo

### In vivo imaging

**Fig. 2:**
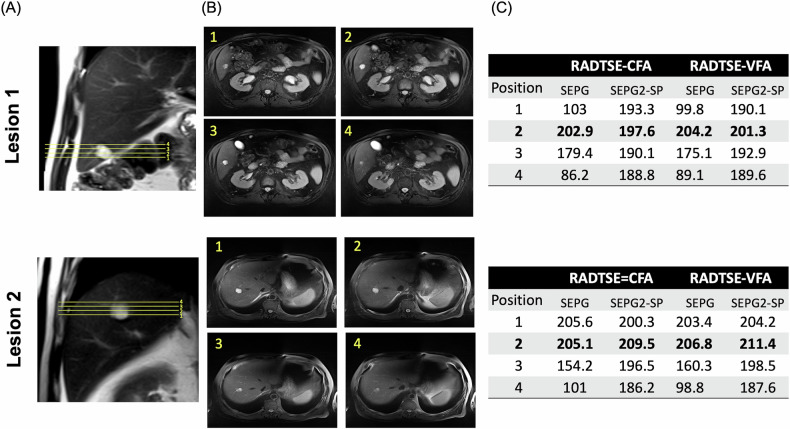
Effect of slice position on lesion T2 estimation: Data were acquired at 3 T using both the RADTSE-CFA and VFA sequences on a subject with two hemangiomas. **A** Coronal images showing the acquired slice positions for each lesion, with at least one position centered on the lesion, where PV is minimized. **B** Axial images for each acquired position; position 2 is centered on the lesion and considered as the position with minimal PV. **C** Estimated T2 values for the SEPG and proposed SEPG2-SP models for the different slice prescriptions

The effect of PV on T2 estimation is illustrated in Fig. 2 for a subject with two small liver hemangiomas (~18 mm in diameter) for data acquired with RADTSE-CFA and RADTSE-VFA. In this experiment, we varied the prescribed slice position with at least one position centered on the lesion as indicated in the coronal (Fig. 2A) and axial (Fig. 2B) orientations. The slice centered in the lesion (position 2 for both lesions) is expected to have minimal PV. With the single-component SEPG model, the T2 values for edge slices (positions 1,3,4) are underestimated compared to the center slice (position 2). In fact, T2 values for positions 1 and 4 for lesion 1 and position 4 for lesion 2 fall in the range for malignancies (see below). With SEPG2-SP, all positions have T2 values closer to the center slice and fall in the range of hemangiomas.

**Fig. 3:**
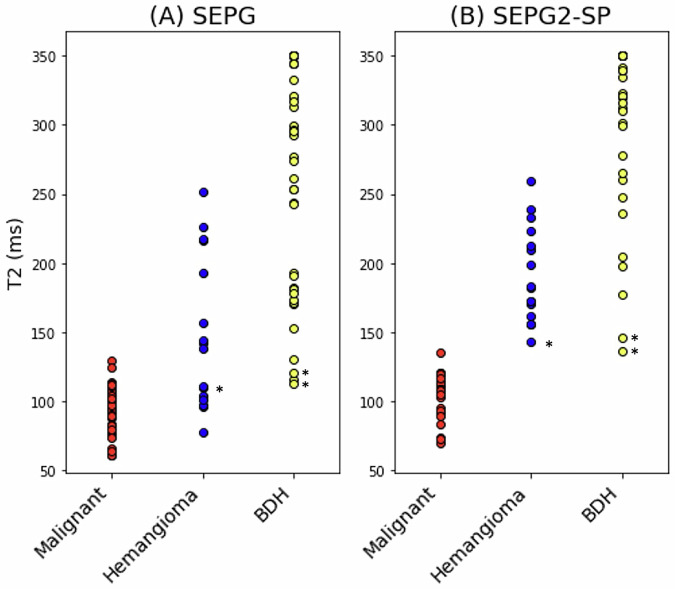
Scatter plots of T2 estimates from data acquired at 1.5 T in 27 clinical patients for a total of 68 neoplasms: 33 malignant (29 metastases, 4 HCC) and 35 benign (7 hemangiomas, 28 bile duct hamartomas (BDH)) lesions comparing (**A**) SEPG (where PV is not considered) to the (**B**) PV-corrected SPG2-SP model proposed here. SEPG has a significant overlap between hemangiomas and malignancies (AUROC = 0.847), with some overlap between BDH and malignancies (AUROC = 0.995). On the other hand, SEPG2-SP provides complete separation between benign and malignant lesions (AUROC = 1.0). The asterisks indicate example lesions shown in Fig. 4

Lesion classification with the SEPG and SEPG2-SP models was evaluated in the clinical dataset comprising the 68 focal liver lesions. For larger lesions spanning more than one slice, T2 was estimated through center and edge slices for a total of 95 ROIs (34 malignant, 16 hemangioma, and 45 BDH). With the single-component SEPG model, the T2 mean and standard deviation for the ROIs were: 92 ± 17 ms (malignancies), 149 ± 55 ms (hemangiomas), and 271 ± 80 ms (BDH). As seen in Fig. 3, there is overlap between malignant and benign lesions, with the overlap driven primarily by the hemangiomas (AUROC = 0.847; specificity = 0.562 at the sensitivity = 1.000 operating point) rather than BDHs (AUROC = 0.995; specificity = 0.933 at the sensitivity = 1.000 operating point).

**Fig. 4:**
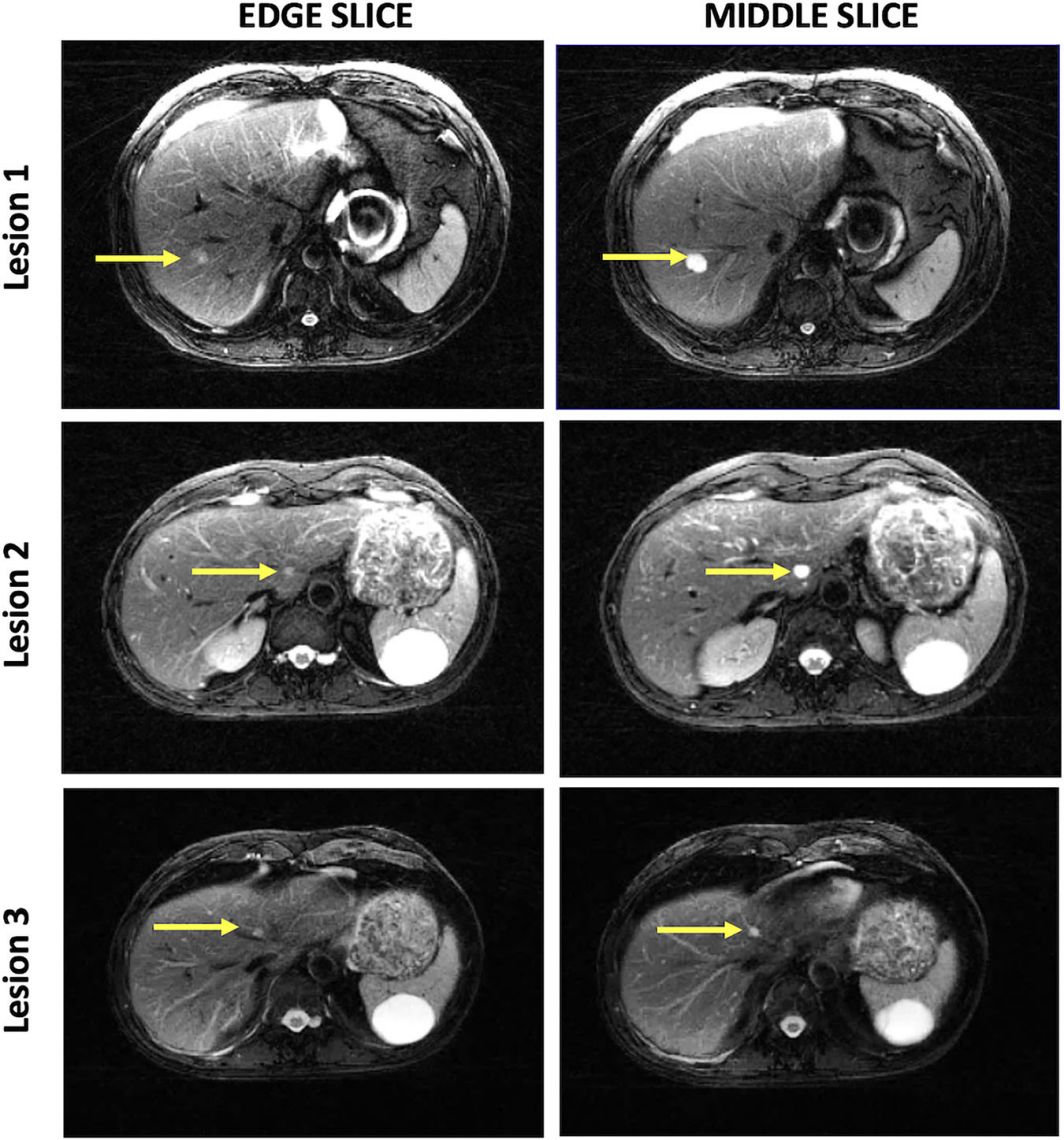
T2-weighted images of three lesions showing the slices at the edge and through the middle of these lesions. The T2 values of ROIs corresponding to the edge slices are shown in Fig. 3 (asterisks)

With the SEPG2-SP model, the T2 mean and standard deviation for the ROIs corresponding to the three lesion types were: 105 ± 15 ms (malignancies), 192 ± 34 ms (hemangiomas), and 312 ± 59 ms (BDH). The SEPG2-SP model classified all lesion ROIs correctly (AUROC = 1.0) regardless of their size or position within the slice. The 3 benign ROIs marked with an asterisk in Fig. 3 are closer to the upper limit of the malignancy region. These correspond to extreme cases where the slice is at the very edge of the lesion, as can be seen in Fig. 4. Even for these extreme cases where the edge slices mimic a very small lesion, the ROIs were classified in the correct category with SEPG2-SP. On the other hand, with the SEPG algorithm, the ROIs fall within the range of malignancies.

## Discussion

We have investigated the utility of RADTSE for the characterization of focal liver lesions using T2 mapping in the presence of PV. RADTSE has several advantages for abdominal imaging. It provides motion-robust T2-weighted images with high spatial resolution, a key feature for detecting small abdominal neoplasms [6]. Furthermore, it provides multiple co-registered TE images acquired with high temporal resolution for adequately sampling the T2 decay curve (32 TE data points sampled every 7.1 ms to cover T2 decay over 227.2 ms) to accurately model the T2 decay, considering the effect of indirect echoes. The multi-component modeling of the T2 signal enables the generation of accurate T2 maps in the presence of PV. The PV-corrected T2 relaxation times were used to correctly classify malignancies and the most common abdominal neoplasms (hemangiomas and BDH), regardless of their size and or position in the imaging slice.

The characterization of small focal liver lesions, which are typically affected by PV, is an important clinical task for precise tumor staging and optimal treatment planning. In colorectal cancer patients, for instance, the detection, anatomic localization and classification of all liver tumors at an early stage with non-invasive imaging is vital to modern management paradigms. Partial volume effects have been shown to introduce a bias in quantitative diffusion imaging, especially for small focal lesions [20, 29]. While recent works in the literature have demonstrated the use of quantitative T2 relaxation time as a biomarker for characterization of abdominal lesions [5–8], they do not consider the effect of partial volume within the voxel that necessitates the use of two-component models. While Kumar et al [24] proposed an algorithm for robust multi-component T2 estimation when using non-180° refocusing flip angles, they do not consider the effect of slice profiles. By modeling both partial volume effects and slice profile imperfections, the proposed two-component model can accurately classify small focal liver lesions.

In turbo spin-echo pulse sequences, the use of a long echo train length (e.g., 32) results in an increase of SAR thereby limiting the number of slices that can be acquired within a given TR [30]. To improve scan efficiency and reduce SAR, we also tested T2 mapping with the variable flip angle RADTSE-VFA sequence. The T2 estimation performance in the presence of PV effects was observed to be comparable between the constant and variable flip angle acquisitions.

While the radial acquisition has an advantage over Cartesian acquisition in terms of motion robustness, it comes at a cost of increased susceptibility to off-resonance and gradient delay effects. The use of fat suppression pulses reduces off-resonance sensitivity. The gradient delays are typically corrected during the pulse sequence design. The results presented here correspond to data acquired with breath-held imaging. The RADTSE pulse sequence could also be used to acquire free breathing data with a prospective navigator for uncooperative subjects [31]. The use of a joint fitting algorithm improves the stability of the estimation, but it increases the computational burden. Most state-of-the-art commercial scanners incorporate GPU-based computing and thus can accommodate the higher computational demand of newer algorithms. The use of deep learning-based algorithms for multi-component modeling could also reduce computational burden [32, 33], and their application to SEPG2-SP should be explored in the future.

## Conclusion

A two-component model accounting for lesion fraction variation within the slice profile is presented. This method improves the characterization of focal liver lesions affected by partial volume yielding correct classification and discrimination of malignant lesions from the most common benign liver lesions based on more accurate T2 measurements. This study provides further evidence that liver tumors may be diagnostically accurately classified using T2 mapping.

## Supplementary information

ELECTRONIC SUPPLEMENTARY MATERIAL

## Abbreviations

AUROC: Area under the ROC curve
BDH: Bile duct hamartomas
EPG: Extended phase graph
PV: Partial volume
RADTSE: Radial turbo spin echo
RADTSE-CFA: Radial turbo spin echo with constant refocusing flip angles
RADTSE-VFA: Radial turbo spin echo with variable refocusing flip angles
ROC: Receiver operating characteristics
ROI: Region-of-interest
SAR: Specific absorption rate
SEPG: Slice resolved extended phase graph
SEPG2-SP: Two-component SEPG model with slice profile variation
TE: Echo time
TSE: Turbo spin echo

## References

[1] Siegelman ES, Chauhan A (2014) MR characterization of focal liver lesions: pearls and pitfalls. Magn Reson Imaging Clin N Am 22:295–313 25086931 10.1016/j.mric.2014.04.005

[2] Mulé S, Kharrat R, Zerbib P et al (2022) Fast T2-weighted liver MRI: image quality and solid focal lesions conspicuity using a deep learning accelerated single breath-hold HASTE fat-suppressed sequence. Diagn Interv Imaging 103:479–485 35597761 10.1016/j.diii.2022.05.001

[3] Cieszanowski A, Anysz-Grodzicka A, Szeszkowski W et al (2012) Characterization of focal liver lesions using quantitative techniques: comparison of apparent diffusion coefficient values and T2 relaxation times. Eur Radiol 22:2514–2524 22699872 10.1007/s00330-012-2519-x PMC3472073

[4] Chen Y, Jiang Y, Pahwa S et al (2016) MR fingerprinting for rapid quantitative abdominal imaging. Radiology 279:278–286 26794935 10.1148/radiol.2016152037 PMC4819902

[5] Szklaruk J, Son JB, Starr BF et al (2020) Evaluation of feasibility and image quality of a new radial quantitative T2 weighted imaging sequence for liver MRI. Clin Imaging 66:77–81 32460150 10.1016/j.clinimag.2020.05.003

[6] Keerthivasan MB, Galons JP, Johnson K et al (2022) Abdominal T2-weighted imaging and T2 mapping using a variable flip angle radial turbo spin-echo technique. J Magn Reson Imaging 55:289–300 34254382 10.1002/jmri.27825 PMC8678192

[7] Xi Lin, Lixing Dai, Qinqin Yang et al (2023) Freebreathing and instantaneous abdominal T2 mapping via single-shot multiple overlapping-echo acquisition and deep learning reconstruction. Eur Radiol 33:4938–4948 36692597 10.1007/s00330-023-09417-2

[8] Fujita S, Sano K, Cruz G et al (2023) MR fingerprinting for contrast agent-free and quantitative characterization of focal liver lesions. Radiol Imaging Cancer 5:e230036 37999629 10.1148/rycan.230036 PMC10698593

[9] Martin DR, Kalb B, Sarmiento JM, Heffron TG, Coban I, Adsay NV (2010) Giant and complicated variants of cystic bile duct hamartomas of the liver: MRI findings and pathological correlations. J Magn Reson Imaging 31:903–911 20373435 10.1002/jmri.22113

[10] Vietti VN, Hilbert T, Bastiaansen JAM et al (2019) Patient respiratory-triggered quantitative T2 mapping in the pancreas. J Magn Reson Imaging 50:410–416 30637852 10.1002/jmri.26612 PMC6766866

[11] Bencikova D, Han F, Kannengieser S et al (2022) Evaluation of a single-breath-hold radial turbo-spin-echo sequence for T2 mapping of the liver at 3T. Eur Radiol 32:3388–3397 34940906 10.1007/s00330-021-08439-y PMC9038820

[12] Erden A, Kuru Öz D, Peker E et al (2021) MRI quantification techniques in fatty liver: the diagnostic performance of hepatic T1, T2, and stiffness measurements in relation to the proton density fat fraction. Diagn Interv Radiol 27:7–14 33290237 10.5152/dir.2020.19654 PMC7837725

[13] Abe Y, Yamashita Y, Tang Y, Namimoto T, Takahashi M (2000) Calculation of T2 relaxation time from ultrafast single shot sequences for differentiation of liver tumors: comparison of echo-planar, HASTE, and spin-echo sequences. Radiat Med 18:7–14 10852650

[14] Altbach MI, Outwater EK, Trouard TP et al (2002) Radial fast spin-echo method for T2-weighted imaging and T2 mapping of the liver. J Magn Reson Imaging 16:179–189 12203766 10.1002/jmri.10142

[15] Huang C, Bilgin A, Barr T, Altbach MI (2013) T2 relaxometry with indirect echo compensation from highly undersampled data. Magn Reson Med 70:1026–1037 23165796 10.1002/mrm.24540 PMC3593752

[16] Crombé A, Buy X, Han F, Toupin S, Kind M (2021) Assessment of repeatability, reproducibility, and performances of T2 mapping-based radiomics features: a comparative study. J Magn Reson Imaging 54:537–548 33594768 10.1002/jmri.27558

[17] Lebel RM, Wilman AH (2010) Transverse relaxometry with stimulated echo compensation. Magn Reson Med 64:1005–1014 20564587 10.1002/mrm.22487

[18] Ben-Eliezer N, Sodickson DK, Block KT (2015) Rapid and accurate T2 mapping from multi-spin-echo data using Bloch-simulation-based reconstruction. Magn Reson Med 73:809–817 24648387 10.1002/mrm.25156 PMC4169365

[19] McPhee KC, Wilman AH (2017) Transverse relaxation and flip angle mapping: evaluation of simultaneous and independent methods using multiple spin echoes. Magn Reson Med 77:2057–2065 27367906 10.1002/mrm.26285

[20] Holzapfel K, Bruegel M, Eiber M et al (2010) Characterization of small (≤10 mm) focal liver lesions: value of respiratory-triggered echo-planar diffusion-weighted MR imaging. Eur J Radiol 76:89–95 19501995 10.1016/j.ejrad.2009.05.014

[21] Huang C, Galons JP, Graff CG et al (2015) Correcting partial volume effects in biexponential T2 estimation of small lesions. Magn Reson Med 73:1632–1642 24753061 10.1002/mrm.25250 PMC4201649

[22] Kumar D, Nguyen TD, Gauthier SA, Raj A (2012) Bayesian algorithm using spatial priors for multiexponential T2 relaxometry from multiecho spin echo MRI. Magn Reson Med 68:1536–1543 22266707 10.1002/mrm.24170

[23] Layton KJ, Morelande M, Wright D, Farrell PM, Moran B, Johnston LA (2013) Modelling and estimation of multicomponent T2 distributions. IEEE Trans Med Imaging 32:1423–1434 23629849 10.1109/TMI.2013.2257830

[24] Kumar D, Siemonsen S, Heesen C, Fiehler J, Sedlacik J (2016) Noise robust spatially regularized myelin water fraction mapping with the intrinsic B1-error correction based on the linearized version of the extended phase graph model. J Magn Reson Imaging 43:800–817 26477610 10.1002/jmri.25078

[25] Prasloski T, Mädler B, Xiang QS, MacKay A, Jones C (2012) Applications of stimulated echo correction to multicomponent T2 analysis. Magn Reson Med 67:1803–1814 22012743 10.1002/mrm.23157

[26] Akhondi-Asl A, Afacan O, Mulkern RV, Warfield SK (2014) T2-relaxometry for myelin water fraction extraction using Wald distribution and extended phase graph. Med Image Comput Comput Assist Interv 17:145–152 25320793 10.1007/978-3-319-10443-0_19 PMC4209472

[27] Pell GS, Briellmann RS, Waites AB, Abbott DF, Lewis DP, Jackson GD (2006) Optimized clinical T2 relaxometry with a standard CPMG sequence. J Magn Reson Imaging 23:248–252 16416434 10.1002/jmri.20490

[28] Hennig J (1988) Multiecho imaging sequences with low refocusing flip angles. J Magn Reson 78:397–407

[29] Hernando D, Zhang Y, Pirasteh A (2022) Quantitative diffusion MRI of the abdomen and pelvis. Med Phys 49:2774–2793 34554579 10.1002/mp.15246 PMC8940741

[30] Keerthivasan MB, Winegar B, Becker JL, Bilgin A, Altbach MI, Saranathan M (2018) Clinical utility of a novel ultrafast T2-weighted sequence for spine imaging. AJNR Am J Neuroradiol 39:1576–1581 30002053 10.3174/ajnr.A5713 PMC7410539

[31] Toner B, Arberet S, Ahanonu E et al (2024) Free-breathing T2 mapping of the abdomen in half the scan time using RADTSE with deep learning reconstruction. In: Proceedings of the annual meeting of the ISMRM Singapore, 0727

[32] Barbieri M, Hooijmans MT, Moulin K et al (2024) A deep learning approach for fast muscle water T2 mapping with subject specific fat T2 calibration from multi-spin-echo acquisitions. Sci Rep 14:8253 38589478 10.1038/s41598-024-58812-2 PMC11002020

[33] Vasylechko SD, Warfield SK, Kurugol S, Afacan O (2024) Improved myelin water fraction mapping with deep neural networks using synthetically generated 3D data. Med Image Anal 91:102966 37844473 10.1016/j.media.2023.102966 PMC10847969

