# Supplementary material for: Diagnosing abdominal neoplasms using a T2 mapping radial turbo spin-echo technique with partial volume correction
Source: Eur Radiol. 2025 Aug 30;36(3):2209–18. doi: 10.1007/s00330-025-11931-4 (PMC12414507; doi:10.1007/s00330-025-11931-4)
Supplement: Supplementary file 1 — ELECTRONIC SUPPLEMENTARY MATERIAL [file 330_2025_11931_MOESM1_ESM.pdf]

Diagnosing Abdominal Neoplasms using a T2 Mapping Radial Turbo Spin-Echo Technique with Partial Volume Correction

ELECTRONIC SUPPLEMENTARY MATERIAL

S1: Signal Modeling

Single component turbo spin-echo signal model:

The observed signal at echo time TE from a turbo spin echo sequence for a single tissue component can be represented using the SEPG model [1]:

$$s(TE) = I \int_z f_{EPG}(T1, T2, B1, \theta_0(z), \theta_1(z) \ldots, \theta_i(z), TE)\, dz \quad [S1]$$

where, $T1$ and $T2$ are relaxation times of the tissue of interest, I is the signal intensity at TE=0, $\theta_0(z)$ is the excitation RF slice profile, $\theta_i(z)$ are the refocusing RF slice profiles, $B1$ is a scaling factor for the excitation and refocusing flip angles and f_{EPG} is the extended phase graph (EPG) signal model [2] describing the signal evolution integrated over the slice profile.

Two component turbo spin-echo signal model:

When there are two tissue types within a slice the observed signal from the slice is dependent upon relaxation of both components. The SEPG signal model from Equation S1 can be extended to include two components:

$$s(TE) = f_{SEPG}(T1_{bg}, T2_{bg}, B1, TE) + f_{SEPG}(T1_{les}, T2_{les}, B1, TE)$$
$$= I_{bg} \int_z f_{EPG}(T1_{bg}, T2_{bg}, B1, \theta_0(z), \theta_1(z) \ldots, \theta_i(z), TE) dz$$
$$+ I_{les} \int_z f_{EPG}(T1_{les}, T2_{les}, B1, \theta_0(z), \theta_1(z) \ldots, \theta_i(z), TE) dz \quad [S2]$$

where the subscripts bg and les denote the background liver and lesion, respectively. I_{bg} and I_{les} are the signal intensities from the background and the lesion, respectively, and determine the lesion fraction. Equation S2 assumes that both tissue types are distributed uniformly throughout the excited slice.

In the case of focal liver lesions, the amount of lesion (i.e., lesion fraction) varies across the slice and the RF slice profile affects the lesion and background differently. To

account for the differences in slice profiles experienced by lesion and background, we can decompose the slice profile into two spatial sub-profiles, Z_{les} and Z_{bg} and the observed signal is obtained by integrating the signal from each component over the sub-profiles. The two-component SEPG model with slice profile variation (SEPG2-SP) can be represented as:

$$s(TE) = \int_{z \in Z_{bg}} f_{EPG}\left(T1_{bg}, T2_{bg}, B1, \theta_0(z), \theta_1(z) \dots, \theta_i(z), TE\right) dz$$

$$+ \int_{z \in Z_{les}} f_{EPG}(T1_{les}, T2_{les}, B1, \theta_0(z), \theta_1(z) \dots, \theta_i(z), TE) dz \quad [S3]$$

$$= f_{SEPG}\left(T1_{bg}, T2_{bg}, B1, \lambda_{bg}, TE\right) + f_{SEPG}(T1_{les}, T2_{les}, B1, \lambda_{les}, TE) \quad [S4]$$

where, λ_{bg} and λ_{les} are slice profile discretization factors for liver and lesion components, respectively. Since the signal from each component is now individually scaled by the sub-profile, the component specific scaling terms (I_{bg} and I_{les}) are no longer necessary.

1. Lebel RM, Wilman AH (2010) Transverse relaxometry with stimulated echo compensation. Magn Reson Med 64(4):1005–14
2. Hennig J (1991) Echoes—how to generate, recognize, use or avoid them in MR-imaging sequences. Part I: Fundamental and not so fundamental properties of spin echoes. Concepts in Magnetic Resonance 3(3):125-43

S2: Image Reconstruction and T2 Estimation

Image Reconstruction: Within a typical breath hold time, we can acquire 192 radial views providing sufficient data to reconstruct an image for each slice with minimal spatial undersampling. This composite image generated using radial views from all the echo times is used as an anatomical reference and has a T2-weighted contrast corresponding to the average of all the acquired TEs. Since in radial TSE all radial views sample the center of k-space, images at the various TEs can also be reconstructed from partial TE data sets generated from the same k-space data used to reconstruct the composite image.

In this work, the under-sampled TE data sets were reconstructed using a subspace constrained iterative reconstruction algorithm [1]. Subspace based techniques [2-6] exploit the signal redundancy across the TE images such that the information in ETL number of images can be well approximated in a lower dimensional subspace. The subspace basis was generated by simulating the TSE signal and the reconstruction problem was solved using 4 principal components to represent the T2 decay. The T2 and B1 ranges for the subspace basis were 20 – 500 ms and 0.6 - 1.3, respectively.

T2 Estimation: Single component T2 maps were generated by fitting the TE images to a library of pre-computed T2 evolution curves [7] based on the SEPG signal model (Equation S1 Supplementary Material). T2 estimation for the two-component model is performed using the joint fitting framework [8]. The model improves the stability of the bi-exponential fit by assuming that the background and lesion T2 values are homogeneous within the lesion's region-of-interest (ROI). Under this assumption, all voxels within the lesion's ROI have two global T2 values for liver and lesion components ($\overline{T2}_{bg}, \overline{T2}_{les}$). This is a valid assumption for hemangiomas and BDH (which are composed of homogenous tissue) and small malignant lesions (small malignancies are less likely to be heterogeneous due to the lack of necrotic regions). The joint fitting algorithm adapted for the SEPG2-SP model is:

$$\widehat{\overline{T2}}_{les}, \widehat{\overline{T2}}_{bg} = \underset{\lambda_{bg}^{1},\ldots,\lambda_{bg}^{M},\lambda_{les}^{1},\ldots,\lambda_{les}^{M},\overline{T2}_{les},\overline{T2}_{bg}}{\arg\min} \sum_{j=1}^{ETL}\sum_{m=1}^{M} \left|\left| f_{SEPG}\left(\overline{T2}_{les}, T1_{les}B1, \lambda_{les}^{m}, TE_j\right) + f_{SEPG}\left(\overline{T2}_{bg}, T1_{bg}, B1, \lambda_{bg}^{m}, TE_j\right) - s_m(TE_j)\right|\right|^2 \quad [S5]$$

where $\lambda_{les}^{1}, \ldots, \lambda_{les}^{M}$ and $\lambda_{bg}^{1}, \ldots, \lambda_{bg}^{M}$ are slice profile scaling factors for the M voxels and $\overline{T2}_{les}, \overline{T2}_{bg}$ are T2 values for lesion and liver within the lesion's ROI.

1. Keerthivasan MB, Galons JP, Johnson K, Umapathy L, Martin DR, Bilgin A, Altbach MI. Abdominal T2-weighted imaging and T2 mapping using a variable flip angle radial turbo spin-echo technique. Journal of Magnetic Resonance Imaging. 2022 Jan;55(1):289-300.

2. Huang C, Graff CG, Clarkson EW, Bilgin A, Altbach MI. T2 mapping from highly undersampled data by reconstruction of principal component coefficient maps using compressed sensing. Magnetic resonance in medicine. 2012 May;67(5):1355-66.
3. Huang C, Bilgin A, Barr T, Altbach MI. T2 relaxometry with indirect echo compensation from highly undersampled data. Magnetic Resonance in Medicine. 2013 Oct;70(4):1026-37.
4. Tamir JI, Uecker M, Chen W, Lai P, Alley MT, Vasanawala SS, Lustig M. T2 shuffling: sharp, multicontrast, volumetric fast spin-echo imaging. Magnetic resonance in medicine. 2017 Jan;77(1):180-95.
5. Mandava S, Keerthivasan MB, Martin DR, Altbach MI, Bilgin A. Improving subspace constrained radial fast spin echo MRI using block matching driven non-local low rank regularization. Physics in Medicine & Biology. 2021 Feb 10;66(4):04NT03.
6. Mandava S, Keerthivasan MB, Li Z, Martin DR, Altbach MI, Bilgin A. Accelerated MR parameter mapping with a union of local subspaces constraint. Magnetic resonance in medicine. 2018 Dec;80(6):2744-58.
7. Huang C, Altbach MI, Fakhri G El. Pattern recognition for rapid T2 mapping with stimulated echo compensation. Magn Reson Imaging. 2014;32(7):969–74
8. Huang C, Galons JP, Graff CG et al (2015) Correcting partial volume effects in biexponential T2 estimation of small lesions. Magn Reson Med 73(4):1632-42

S3: Simulation Experiments

Monte-Carlo simulations were conducted to evaluate the performance of the SEPG2-SP model. A numerical phantom was constructed, assuming a spherical lesion embedded within a background representing liver tissue. The lesion and background pixels were assigned the following relaxation times based on a normal distribution at 3T: T1=1400$\pm$50 ms, T2=180$\pm$5 ms for hemangioma (lesion) and T1=600$\pm$30 ms and T2=40$\pm$5 ms for liver. The slice profiles corresponding to the RF pulses used in RADTSE were generated using the Shinnar–Le Roux algorithm [1] assuming an excitation and refocusing slice thickness of 10 mm, 8mm, and 6mm. The signal at each voxel was simulated using the SEPG model while accounting for the slice profiles experienced by the two different species. TE images were generated from the forward model and independent and

identically distributed Gaussian noise was added to the images. T2 values were estimated by fitting the simulated data to a single component SEPG model and the two component SEPG2-SP model using the corresponding joint estimation algorithm. The mean and standard deviation of the estimated T2 values were computed from 100 noise realizations for each fitting approach. T2 estimation accuracy was evaluated by computing the relative error with respect to the reference estimate ($T2_{REF} = 180\ ms$):

$$Relative\ Error\ (\%) = \frac{|T2_{REF} - T2\ \ |}{T2_{REF}} * 100$$

1. Pauly J, Le Roux P, Nishimura D, Macovski A. Parameter relations for the Shinnar-Le Roux selective excitation pulse design algorithm (NMR imaging). IEEE transactions on medical imaging. 1991 Mar;10(1):53-65.

S4: Phantom Imaging Experiments

A physical phantom was prepared to evaluate the effect of PV in T2 estimation (Figure S1). Three NMR tubes ending in a spherical bulb were filled with a 0.14 mM gadobenate dimeglumine (Multihance^{TM}, Bracco Diagnostic Inc) solution resulting in T2 = 160 ms and T1 = 1090 ms (to represent the T2 of a hemangioma). The diameters of the spherical bulbs were 8 mm, 9.5 mm and 12 mm as measured from a 0.54 mm x 0.54 mm spin echo image. The bulbs were immersed in a container filled with a 3.6 mM gadobenate dimeglumine solution (T2 = 44 ms, T1 = 60 ms) to represent spherical lesions embedded in background tissue. Data were acquired with both the RADTSE-CFA and RADTSE-VFA pulse sequences with the following parameters: FOV = 14 cm, base resolution = 256, radial views = 256, ETL = 32, echo spacing = 6.8 ms. Slice thickness 10 mm was used to ensure the excited slice covers the smallest phantom sphere. Data were acquired with slices prescribed with 6mm, 4mm, and 0mm relative offsets from the center of the spherical bulb to introduce varying levels of PV. Reference T2 estimates were obtained by scanning the spherical bulbs and background tubes separately using a single-echo spin-echo pulse sequence with the following parameters: FOV= 14 cm, acquisition matrix = 256x256, TR = 5000 ms, slice thickness = 5 mm, and 16 different TEs from 10 ms – 320 ms.

T2 estimation accuracy in phantoms was evaluated by computing the relative error with respect to the single-echo spin echo reference ($T2_{SE}$):

$$Relative\ Error\ (\%) = \frac{|T2_{SE} - T2_{RADTSE}|}{T2_{SE}} * 100.$$

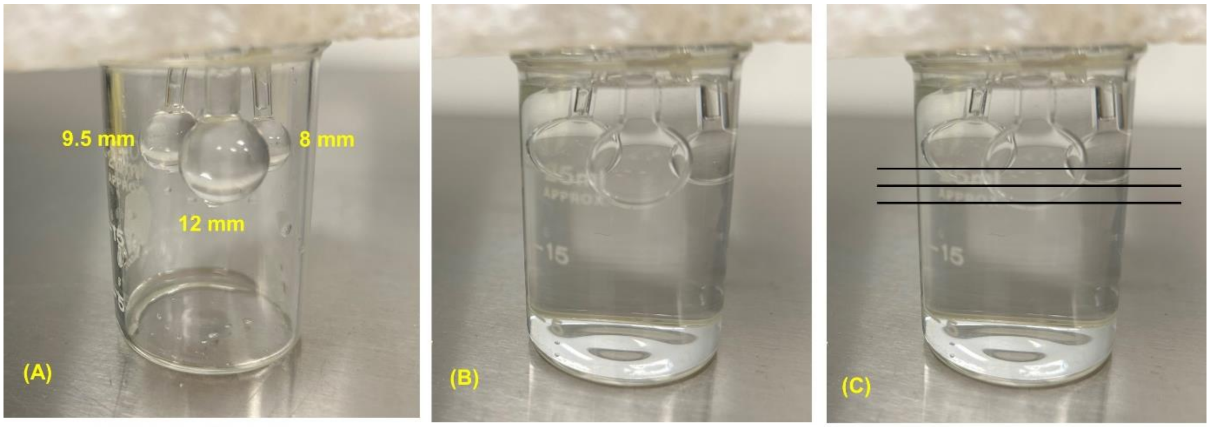

NMR bulbs filled with
0.14 mM MultiHance solution
(T2~160 ms)

NMR bulbs embedded in a
0.36 mM MultiHance solution
(T2~44 ms)

Figure S1: Physical phantom used for mimicking partial volume (PV) effects on spherical lesions embedded in a background. (A) RADTSE data were acquired on the NMR spherical bulbs filled with 0.14 mM MultiHance to determine the T2 of the bulbs without PV effects. (B) A 0.36 mM MultiHance solution was added to the beaker to mimic liver as background. (C) RADTSE data were acquired on the phantom in B with the slice location (black line) prescribed at different offsets from the center of each bulb to have different degrees of PV.
